# The Effectiveness of Dietary Byproduct Antioxidants on Induced CYP Genes Expression and Histological Alteration in Piglets Liver and Kidney Fed with Aflatoxin B1 and Ochratoxin A

**DOI:** 10.3390/toxins13020148

**Published:** 2021-02-15

**Authors:** Roua Gabriela Popescu, Cristina Bulgaru, Arabela Untea, Mihaela Vlassa, Miuta Filip, Anca Hermenean, Daniela Marin, Ionelia Țăranu, Sergiu Emil Georgescu, Anca Dinischiotu

**Affiliations:** 1 Department of Biochemistry and Molecular Biology, Faculty of Biology, University of Bucharest, Splaiul Independentei No. 91–95, 050095 Bucharest, Romania; roua.popescu@drd.unibuc.ro (R.G.P.); anca.dinischiotu@bio.unibuc.ro (A.D.); 2 Laboratory of Animal Biology, National Institute for Research and Development for Biology and Animal Nutrition, Calea Bucuresti No. 1, Balotesti, 077015 Ilfov, Romania; cristina.bulgaru@ibna.ro (C.B.); arabela.untea@ibna.ro (A.U.); daniela.marin@ibna.ro (D.M.); 3 Raluca Ripan Institute for Research in Chemistry, Babeş Bolyai University, 30 Fântânele Street, 400294 Cluj-Napoca, Romania; mihaela.vlassa@ubbcluj.ro (M.V.); miuta.filip@ubbcluj.ro (M.F.); 4 “Aurel Ardelean” Institute of Life Sciences, Vasile Godis Western University of Arad, Rebreanu 86, 310414 Arad, Romania; anca.hermenean@gmail.com

**Keywords:** piglets, antioxidant effect, feed additives, mycotoxins, CYPs gene expression

## Abstract

The purpose of this study was to investigate the potential of a byproduct mixture derived from grapeseed and sea buckthorn oil industry to mitigate the harmful damage produced by ochratoxin A and aflatoxin B1 at hepatic and renal level in piglets after weaning. Forty cross-bred TOPIGS-40 hybrid piglets after weaning were assigned to three experimental groups (E1, E2, E3) and one control group (C), and fed with experimental diets for 30 days. The basal diet was served as a control and contained normal compound feed for starter piglets without mycotoxins. The experimental groups were fed as follows: E1—basal diet plus a mixture (1:1) of two byproducts (grapeseed and sea buckthorn meal); E2—the basal diet experimentally contaminated with mycotoxins (479 ppb OTA and 62ppb AFB1); and E3—basal diet containing 5% of the mixture (1:1) of grapeseed and sea buckthorn meal and contaminated with the mix of OTA and AFB1. After 4 weeks, the animals were slaughtered, and tissue samples were taken from liver and kidney in order to perform gene expression and histological analysis. The gene expression analysis showed that when weaned piglets were fed with contaminated diet, the expression of most analyzed genes was downregulated. Among the CYP450 family, *CYP1A2* was the gene with the highest downregulation. According to these results, in liver, we found that mycotoxins induced histomorphological alterations in liver and kidney and had an effect on the expression level of *CYP1A2*, *CYP2A19*, *CYP2E1*, and *CYP3A29*, but we did not detect important changes in the expression level of *CY4A24*, *MRP2* and *GSTA1* genes.

## 1. Introduction

Mycotoxins are secondary toxic metabolites produced by certain strains of filamentous fungi. These low molecular weight compounds (up to 500 Da) can contaminate a variety of raw materials and cause an increased risk to human and animal health [1]. The number of mycotoxins characterized and with well-known effects is relatively small due to the multitude of metabolites with toxic potential generated by fungi [2,3,4]. They are classified into five groups, with specific chemical structures that occur frequently in feed and food: trichothecenes, zearalenone, ochratoxins, fumonisins, and aflatoxins. The mycotoxins producing fungi found in food and feed are divided into two groups: those that invade before grain harvesting, called field fungi, and those that grow only after harvesting, called storage fungi [5]. At the European level, there are regulations and recommendations regarding the maximum accepted level for six types of mycotoxins commonly found in pigs’ feed: aflatoxins, fumonisins, ochratoxins, deoxynivalenol, T2 toxin, and zearalenone [6,7,8].

Among the farm animal species, pigs are very sensitive to mycotoxins due to their exposure to cereal-based fodders [9]. Swine metabolism is not effective in detoxifying and excreting mycotoxins, which increases the risk of mycotoxicosis. This susceptibility also varies with age, concentration of mycotoxins in feed, and duration of exposure. Liver is organ most affected by the ingestion of these toxins [10]. Furthermore, these toxins increase the permeability of the intestinal epithelial barrier in swine and poultry, which could generate predisposition for necrotic enteritis [11] and the decrease of innate immunity.

Aflatoxins represent the most abundant mycotoxins found in foodstuffs, oilseeds, cereals, milk, soils, animals, and humans. All types of aflatoxins are derived from fungal species belonging to the genus *Aspergillus* and are considered among the most harmful mycotoxins for animals and humans [4,10,11,12,13,14,15,16,17]. As mentioned above, in suckling piglets and growing, finished, and breeding pigs, the main biological effects of aflatoxins are carcinogenicity, immunosuppression, mutagenicity, teratogenicity, decreased feed efficiency and poor weight gain, impaired liver, and altered serum biochemical parameters [18,19]. Severe effects in swine can lead to acute hepatitis, systemic hemorrhages, nephrosis, and death [20], as well as decreased resistance to stress [21]. Some authors have also shown that swine fed with low levels of aflatoxins presented signs of pulmonary edema, reduced feed consumption and body weight gain, and a decrease in the enzymatic activities implicated in oxidative decarboxylation, as well as total serum protein, blood pressure, and total leukocyte count [18,22,23,24]. In this context, according to the European Commission Directive 2003/100/EC, the maximum aflatoxin B1 (AFB1) accepted level for pigs is set at 0.02 mg/kg.

Ochratoxins are secondary metabolites produced by fungal species belonging to the genus *Aspergillus* and *Penicillium*. Divergent opinions regarding the genotoxic or nongenotoxic mechanisms of ochratoxins toxicity have been published [25,26]. In vitro and in vivo studies revealed that guanine-OTA-specific DNA adducts persisted for more than 16 days at renal level, whereas in liver and spleen, they were removed after 5 days [27]. Due to this, their main toxic and carcinogenic effects were exerted in kidney [28].

Most metabolites of ochratoxins from Phase I and Phase II detoxification have low toxicity. In the stomach, a part of ochratoxins is hydrolyzed to ochratoxin α by proteolytic enzymes. Another possibility for their hydrolysis is the opening of the lactone ring under alkaline conditions of intestine, thus resulting in a compound with high toxicity. Due to the strong binding to albumin, the elimination of ochratoxins by glomerular filtration is negligible, with the excretion being mainly through tubular secretion. The tubular resorption is considered partially responsible for the intracellular accumulation of ochratoxins [29,30].

Generally, in farm animals, ochratoxins are rapidly absorbed after ingestion through the gastrointestinal tract (stomach and proximal portion of the jejunum) in a passive manner, which is favored by the high affinity of binding of ochratoxins to plasma proteins, and in a nonionized form, which explains their persistence in the body. In porcine serum, ochratoxins bind more specifically to proteins with a molecular mass less than 20 kDa, allowing them to pass through the glomerular basement membrane and exert nephrotoxic effects. Ochratoxins also accumulate in liver and muscles. However, kidneys are the main site of ochratoxins storage, with their reabsorption at the proximal and distal tubules contributing to the body persistence and increased nephrotoxicity [27,31].

On the other hand, once AFB1 is absorbed at the intestinal level, it reaches liver where it is transformed by Phase I metabolizing enzymes by hydroxylation, hydration, demethylation, and epoxidation. The first three reactions generate nontoxic metabolites, whereas the fourth produces AFB1-8,9 epoxide that forms adducts with DNA at the N7 site of guanine [32]. Also, AFB1 can be conjugated with reduced glutathione in a reaction catalyzed by glutathione-S-transferases [33] and glucuronic acid [34]. Excretion of AFB1 occurs primarily through the biliary pathways, followed by the urinary pathway [35].

One of the main difficulties encountered in controlling mycotoxins is that more than one type of mycotoxin is present in a batch of fodder or cereal at the same time. Thus, feeding of piglets and pigs with contaminated feed with several types of mycotoxins, even if they are in minimum concentrations, can cause numerous negative consequences due to their synergistic effect [36,37,38,39,40]. In this context, diminishing and eliminating the negative effects of mycotoxins found in swine feed could decrease production cost and loss in the pig industry.

To date, numerous strategies have been developed to prevent, reduce, or even eliminate mycotoxin contamination from animal feed by biological, chemical, and physical detoxification methods. These methods allow the degradation of mycotoxins and their corresponding metabolites and maintain the nutritional value of the food without introducing other substances with toxic potential into the biological systems [6,14,41].

Biological decontamination of mycotoxins using competitive inhibition by other fungi strains or addition of antioxidant compounds in animal feed in order to reduce the toxic effects of mycotoxins and/or to inhibit the growth of mycotoxin-producing fungus species represents a good solution. The most used method to counteract the negative impact of mycotoxins on farm animals is adding “mycotoxin binders” or “mycotoxin modifiers,” which are aluminosilicates with a porous structure that are able to adsorb and trap mycotoxins [42,43,44]. They are very effective for aflatoxins and have limited activity against other types of mycotoxin. However, being nonspecific, they also bind vitamins and trace elements, generating deficiencies [45,46,47]. Adding some plant-derived antioxidants in feed could be a better solution [48] to diminish the deleterious effects of mycotoxins on animal health.

P450 cytochromes enzymes, mainly present in liver, intestinal tract, and kidney, play an important role in phase I biotransformation of xenobiotics, especially those belonging to the families 1 and 3 [49]. Mycotoxins can be substrates, inhibitors, or inducers of these metabolizing enzymes. Changes in the specific activity and inducibility of cytochromes P450 will ultimately determine the relative change in the metabolism of a xenobiotic. Mycotoxins may alter the gene expression of these proteins, leading to an altered absorption and biotransformation of nutrients and other substrate drugs from feed. Due to this, the aim of the present study was to investigate the potential of a byproduct mixture derived from *Vitis vinifera* (grapeseed) and *Hippophae rhamnoides* (sea buckthorn) oil industry to mitigate the harmful damage produced by the concomitant presence ochratoxin A (OTA) and aflatoxin B1 (AFB1) in feed at the hepatic and renal level in piglets after weaning.

## 2. Results

### 2.1. Diet Composition

The chemical composition of byproducts meal showed that sea buckthorn meal is richer in protein (+38.4%), fat (+66.6%), and carbohydrates and lower in ash than grapeseed meal (Table 1).

The chemical analysis also showed a different profile of the two byproducts in fatty acids, flavonoids, phenolic acids, and minerals. Thus, the sea buckthorn meal has a higher content of saturated fatty acids (palmitic and palmitoleic), omega-9 acids (cis oleic acid), and omega-3 acids (α-linolenic acid) than the grapeseed meal. In contrast, the grapeseed meal has a very high omega-6 acids (linoleic acid) content (67.35% compared to 18.59% in sea buckthorn meal) (Table 2).

Both byproducts contain flavonoids and phenolic acids, bioactive compounds known for their antioxidant, anti-inflammatory and immunomodulatory properties [50,51]. Thus, the total concentration of polyphenols was 74.8% higher in grapeseed meal (133.84 mg GAE/L) than in sea buckthorn (76.57 mg GAE/L). Concerning the different classes of polyphenols, grapeseed meal contains higher concentration of catechin and vanillic acid than sea buckthorn, while sea buckthorn is richer in rutin, quercitrin, luteolin, p-coumaric acid, and ferulic acid (Table 3).

Regarding the mineral composition, sea buckthorn meal shoeds a higher content of K, Mg, Fe, Mn, and Zn than grapeseed meal. In contrast, grapeseed meal contained twice as much copper as sea buckthorn meal. Of note is the high concentration of iron from sea buckthorn meal (Table 4).

### 2.2. Animal Performance

Exposure of piglets from E2 group to ochratoxin plus aflatoxin B1 mixture had no adverse effects on body weight, weight gain, and feed intake, as the differences were not significant compared to the control. In contrast, the administration of the diet containing the byproducts mixture alone (E1) increased significantly the body weight of piglets fed this diet when compared to control (32.14 ± 1.63 vs. 27.09 ± 1.31) and to group E2, which was fed the contaminated diet (32.14 ± 1.63 vs. 28.72 ± 1.07). It should be noted that the group of piglets receiving contaminated feed and the mixture of byproducts had a tendency to gain weight compared to the group of mycotoxin-intoxicated piglets, although the difference was not significant. Biochemical parameters analysis, which characterizes the general state of animal health and the functionality of liver and kidneys, registered normal values for the age and weight category of weaned piglets. No significant differences were identified between groups for most of them (Table 5). However, the mycotoxin mixture increased ALP and gamma GT activity compared to control and decreased activity in the control level in group E3 receiving the byproduct mixture.

### 2.3. Histology of Liver and Kidney

**Figure 1:**
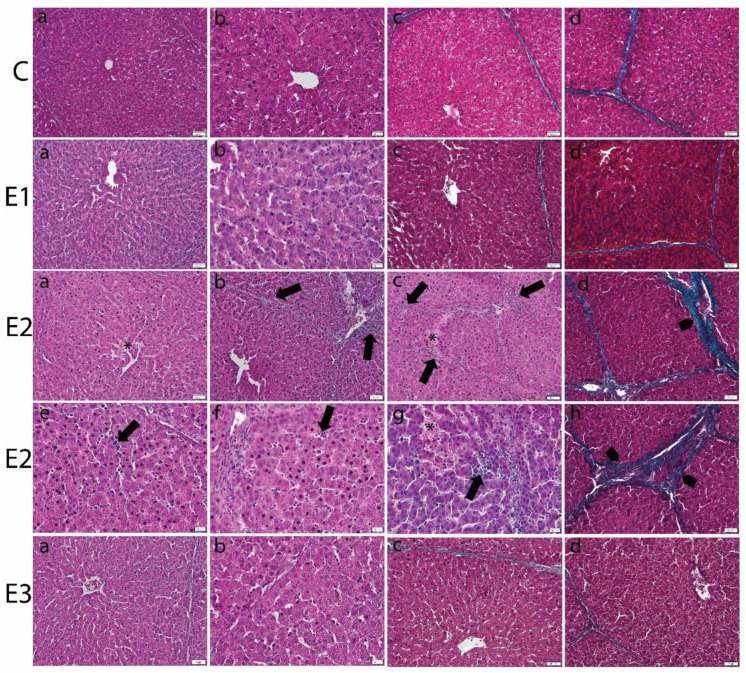
Histopathological changes in liver of weaned piglets subjected to experimental diets. The Control group (C) showed the normal aspect of hepatocytes and sinusoids (**a**,**b**) in the H&E stain and the normal aspect of thin perilobular (**c**) and priportal (**d**) fibrous spikes in Gomori’s trichrome stain. The E1 group showed the normal aspect of the liver in the H&E stain (**a**,**b**) and Gomori’s trichrome stain (**c**,**d**). The E2 group showed dilated sinusoids and inflammatory infiltrates (arrows) and necrotic hepatocytes (*) in the H&E stain (**a**–**c**,**e**–**g**), and perilobular (**d**) and priportal (**h**) fibrosis (arrowhead) in Gomori’s trichrome stain. The E3 group displayed marked improvement of the histological aspect of the liver, which is comparable to that of the control group, in the H&E (**a**,**b**) and Gomori’s trichrome stains (**c**,**d**). Scale bar = 50 μm.

Light microscopic analysis of the livers from E2 group, fed with a basal diet contaminated with a mixture of OTA and AFB1, showed focal areas of necrosis, dilatation of sinusoid, and inflammatory parenchymal infiltration. The portal areas revealed mononuclear cellular infiltration and periportal fibrosis. The fibrotic perilobular fibrotic septa were also noticed (Figure 1).

**Figure 2:**
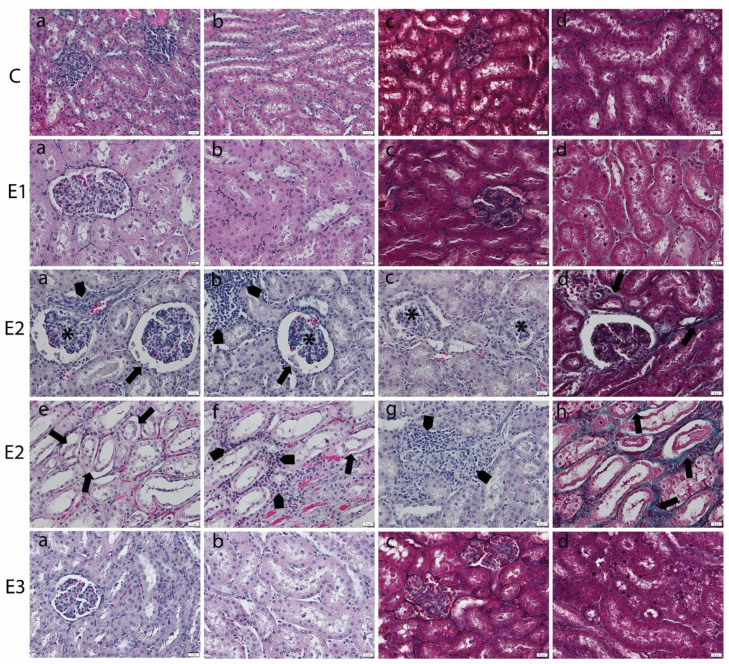
Histopathological changes in kidney of weaned piglets subjected to experimental diets. The Control group (C) showed the normal aspect of kidney cortex (**a**) and medulla (**b**) in the H&E stain and few collagen fibers surrounding glomeruli and tubules in cortex (**c**) and medulla (**d**) in Gomori’s trichrome stain. The E1 group showed the normal aspect of kidney cortex (**a**) and medulla (**b**) in the H&E stain and few collagen fibers surrounding glomeruli and tubules in the cortex (**c**) and medulla (**d**) in Gomori’s trichrome stain. The E2 group (**a**–**d**) kidney cortex showed glomerular atrophy (*), Bowmann’s capsule injury (arrow), inflammatory cell infiltrates (arrowhead) (**a**,**b**), or glomerular degeneration (*) in the H&E stain (**a**–**c**) and slight proliferation of peritubular collagen in Gomori’s trichrome stain (arrow) (**d**). (**e**–**h**) The kidney medulla showed altered tubuli (arrow), inflammatory infiltrates (arrowhead), and congestion in blood vessels in the H&E stain (**e**–**g**) and the proliferation of peritubular collagen in Gomori’s trichrome stain (arrow) (**h**). The E3 group displayed marked improvement of the renal histological aspect, which is comparable to that of the control group, in the kidney cortex (**a**) and medulla (**b**) in the H&E stain and in the cortex (**c**) and medulla (**d**) in Gomori’s trichrome stain. Scale bar = 50 μm.

Mycotoxin administration caused structural changes in kidneys that affected both the cortex and medulla. Atrophy of the glomerular tufts and alteration of the Bowmann’s capsule were noticed (Figure 2). The tubules showed necrosis of lining epithelial cells with inflammatory cells infiltration in between. Focal aggregates of inflammatory cells were observed in between the glomeruli and tubules in association with the focal areas of congestion in blood vessels, especially in the medullary region. Apparently, the collagen proliferation was mainly observed in areas of tubular injury. Furthermore, kidney sections from the E3 groups, the group fed with a basal diet containing a mixture of grapeseed and sea buckthorn meal and contaminated with the mix of OTA and AFB1, revealed minor pathomorphological changes, almost similar to control.

Moreover, the morphometric analysis of the structural injuries in liver and kidney of experimental groups was evaluated (Table 6).

### 2.4. The Level of Gene Expression

**Figure 3:**
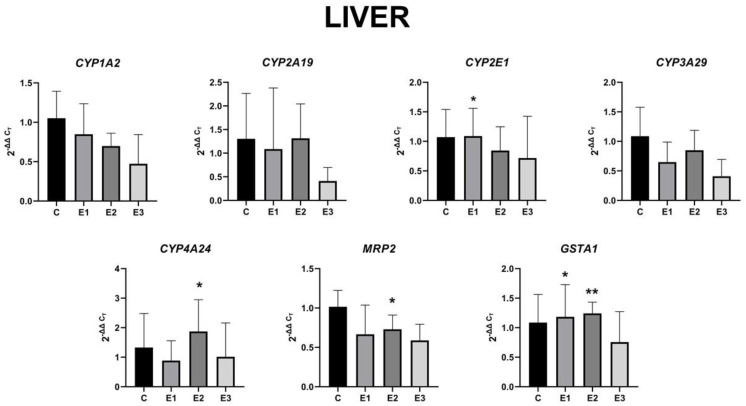
Gene expression level in the liver for *CYP1A2*, *CYP2A19*, *CYP2E1*, *CYP3A29*, *CYP4A24*, *MRP2*, and *GSTA1* of weaned piglets subjected to experimental diets. The data are illustrated as average values of the groups (n = 4) ± standard deviation of the mean (STDEV). Statistical significance: * *p* < 0.05; ** *p* < 0.01. The statistical significance of the changes is related to the control group level.

We found that modifying the piglets’ diet caused significant liver changes to the *CYP2E1* and *GSTA1* genes in the E1 group fed with a basal diet supplemented with a mixture of grapeseed and sea buckthorn meal, and to the *CYP4A24*, *MRP2*, and *GSTA1* genes in the E2 group fed with a basal diet contaminated with a mixture of AFB1 and OTA. The modifications caused insignificant changes to all the other target genes (Figure 3).

In liver, the gene expression for *CYP1A2* decreased by 18% for E2 and 44% for E3, respectively, compared to the E1 group. The *CYP2A19* gene expression was unmodified in groups E1 and E2, whereas in group E3, it decreased by almost 62%. A significant increase by 29% was observed in *CYP2E1* gene expression in the E1 group fed with a basal diet supplemented with a mixture of grapeseed and sea buckthorn meal compared to the E2 group. In contrast, the administration of basal diet enriched with a mixture of grapeseed and sea buckthorn meal (E1 group) downregulated the *CYP3A29* gene expression by 24% compared to the E2 group level. Another contrast was observed in the *CYP4A24* gene expression, with a 33% decrease for the E1 group and 24% decrease for the E3 group, and a significant 41% increase in the E2 group fed with a basal diet supplemented with a mixture of AFB1 and OTA, compared to the control level. In the case of *MRP2*, the gene expression pattern was similar to that of the *CYP4A24* gene, with an insignificant 35% decrease for the E1 group and 24% decrease for the E3 group, and a significant 28% increase in the E2 group, compared to the control level. Similarly, to the *CYP4A24* gene expression, the *GSTA1* gene expression showed a significant 14% increase in the E2 group, a 9% increase in the E1 group, and a 30% decrease for the E3 group. Obviously, the concomitant administration of the mixture of grapeseed and sea buckthorn meal and OTA and AFB1 generated a decrease of all analyzed genes expressions in liver compared to control.

**Figure 4:**
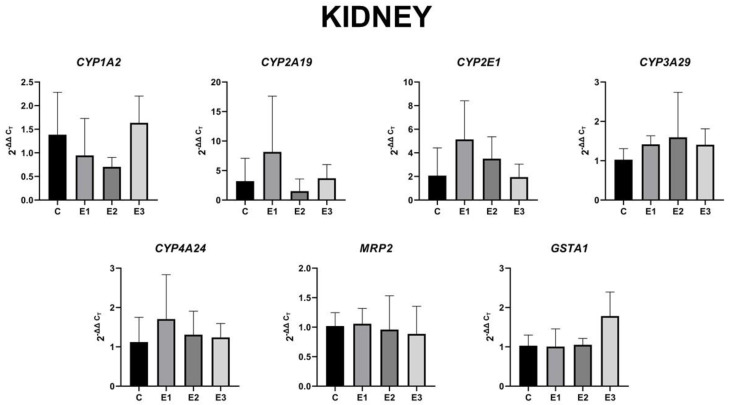
Gene expression level in the kidney for *CYP1A2*, *CYP2A19*, *CYP2E1*, *CYP3A29*, *CYP4A24*, *MRP2*, and *GSTA1* of weaned piglets subjected to experimental diets. The data are illustrated as average values of the groups (n = 4) ± standard deviation of the mean (STDEV).

Regarding the expression level of these genes in kidneys, compared to liver samples, no statistically significant changes were observed (Figure 4). However, changes in the regulation of gene expression level could be observed.

Analyzing Figure 4, it could be noticed that the mixture of grapeseed and sea buckthorn meal downregulated the *CYP1A2* gene expression and upregulated the *CYP2A19, CYP2E1, CYP3A29*, and *CYP4A24* gene expression in an insignificant way, whereas *MRP2* and *GSTA1* gene expression remained unmodified. Also, the presence of OTA and AFB1 in piglets feed downregulated *CYP1A2* and *CYP2A19* gene expression in an insignificant way, whereas *MRP2* and *GSTA1* were unmodified. The concomitant administration of the mixture of grapeseed and sea buckthorn meal and OTA and AFB1 determined the return of all genes expression levels to control levels with the exception of *GSTA1*, which presented an important increase compared to E1 group.

## 3. Discussion

Mycotoxins such as AFB1 and OTA are natural toxins contaminating a large variety of plant products. As a consequence, AFB1, OTA, and their metabolites are present in food and feed, as well as in the products of animal origin [52]. Most of the toxicological studies regarding the effects of mycotoxins have considered the exposure to a single type of mycotoxin without considering the combination and the interaction between them, respectively, the synergistic or antagonistic effects which often occur in nature. Data regarding the toxic effects of mycotoxin combinations are limited, so the risks of exposure to several types of toxins are still unknown.

The occurrence of mycotoxins such as AFB1, DON, ZEA, OTA, FB1, and FB2 in cereal, cereal products, and complementary and complete feeding stuffs for pigs [16] is related to the geographical location and climate change, which increases the risk associated with mycotoxin contamination during the storage and processing of feed products for pigs [53]. The co-contamination of cereals and other raw materials occurs more frequently in real life than single mycotoxin contamination [7]. For example, the co-occurrence of aflatoxin B1 and ochratoxin A has been found in different food or feed ingredients, such as wheat [54], barley [55], cereal flours [56], spice [57], etc. The proportion between AFB1 and OTA in feed was found to be about 1 to 6 [37]. Also, the global feed content in AFB1 and OTA ranged between not determined and 100 ppb and not determined and 211 ppb, respectively [58]. In this context, in order to mimic the field conditions, we studied the effects of these mycotoxins together and to assess the effectiveness of the by-product mix in counteracting the effects of mycotoxins. The natural additives (grapeseed and sea buckthorn byproducts) were selected based on their ability to ameliorate mycotoxicosis upon dietary supplementation [59,60].

In the present study, the exposure of piglets (E2 group) to mycotoxins mixture did not influence the performance of animals (27.83 ± 1.1 vs. 27.09 ± 1.3 for body weight and 1.48 ± 0.9 vs. 1.40 ± 0.8 for feed intake) and biochemical parameters when compared to control. Similarly, Balogh et al. [61] reported that piglets fed with approximately 0.4 mg/kg of OTA during the starter (0–28 days) and grower (29–49 days) period did not register significantly changes in the production traits and clinical signs of toxicity in the grower phase. In contrast, a significant decrease of body weight gain was observed during the starter period when the animals were more sensitive. In this study, the dietary inclusion of the byproduct mixture alone had a significant influence on animal performance (group E1) and tended to increase piglets’ weight when the mixture was associated with contaminated food (group E3).

From a toxicological point of view, OTA is classified by IARC (International Agency for Research on Cancer) in the same group (2B) of carcinogenic substances for humans, having a similar toxicity with AFB1 [62]. Toxicokinetic patterns of absorption, distribution, and elimination for these mycotoxins are, for the most part, entirely elucidated. In contrast, despite recent progress, our knowledge of the toxicokinetic biotransformation steps is not elucidated in detail. A number of studies have shown that AFB1 and OTA are metabolized by liver microsomes from humans, pigs, and rats into several epimers [63]. Changes in the specific activity and inducibility of cytochromes P450 ultimately determine the relative change in the metabolism of any xenobiotic.

It has been found that exposure to AFB1 and OTA decreased the gene expression of *CYP1A2*, *CYP2E1*, *CYP3A29,* and *MRP2* genes in pig’s liver and resulted in several changes in liver histology and ultrastructure, including focal areas of necrosis, dilatation of sinusoid, inflammatory parenchymal infiltration, and periportal fibrosis. Regarding the gene expression level of CYP450 isoforms in pig’s kidney, no data were available in the scientific literature.

The *CYP1A2*, *CYP2A19*, *CYP2E1*, *CYP3A29*, *CYP4A24*, *MRP2*, and *GSTA1* genes were chosen for this study because they encode proteins with enzymatic activity or transporter function that are involved in Phase I and Phase II of biotransformation and detoxification of xenobiotics to form electrophilic reactive metabolites [64].

According to these results, it appears that the by-product administration determined a decrease in *CYP1A2* gene expression and an increase in *GSTA1* gene expression. Similar results were noticed in HT-29 human colon cancer cells treated with *Salicornia freitagii* extract, known for its antioxidant and anti-inflammatory activity. In this case, due to its content in bioactive phenols, a downregulation of *CYP1A2* mRNA and an upregulation of GSTA1 mRNA occurred [65]. In contrast to our results, mRNA and protein expression of *CYP1A2* were increased in liver of chicory fed pigs [66]. These different results were probably caused by the different natural compounds present in chicory compared to the byproducts used in the present study, mainly chlorogenic, caffeic, and p-coumaric acids [67].

On the other hand, OTA and AFB1 probably interacted with and activated the aromatic hydrocarbon receptor, leading to its nuclear translocation. After the heterodimerization, OTA and AFB1 probably interacted with hydrocarbon receptor nuclear translocator, the heterodimer, bound to xenobiotic-responsive elements and transactivated genes such as *CYP1A1*, *CYP1A2*, and *GST* [68]. This xenobiotic-responsive element is shared between *CYP1A1* and *CYP1A2* genes [69], and the two enzymes codified by them present overlapping substrate specificity [70]. In pig liver, only *CYP1A2* activity is present, and its relative amount of total detected CYP450 is 4% [71]. In the human liver, AFB1 and OTA are inducers for CYP1A1, 1A2, 2B6, 2C9, 3A4, and 3A5 [72]. AFB1, as well as OTA exposure, generate mitochondrial dysfunction characterized by an increase in ROS production [14] that could increase TGF-β1 expression or activate latent TGF-β1 [73]. Taking into consideration, the previous evidence that TGF-β1 decreased *CYP1* expression in humans and rats, it is possible that the same mechanism [74] occurred under our conditions. The effects of the concomitant exposure to both mycotoxins and grapeseed and sea buckthorn by-products were probably synergistical, and the expression of *CYP1A2* was lower in E3 compared to E1, E2, and the control group. *CYP1A2* is expressed in lower levels in extrahepatic tissues [75].

The kidney is an organ that receives about 25% of cardiac output and purifies metabolic residue and xenobiotics from the circulatory system. During this discharging process, toxic substances are concentrated in the kidney [76]. In piglet kidneys, the variation of *CYP1A2* gene expression was similar with the expression levels in liver for E1 and E2. Interestingly, in the E3 group, the expression of this gene was at a higher level than the control group. This could possibly be due to the activation of noncanonical signaling pathway for AhR transcription in the kidney cells [77].

In pig liver, the relative amounts of *CYP2A19* and *CYP2E1* represent 31% respectively 13% of total CYP450 [71]. Porcine *CYP2A19* and *CYP2E1* genes are responsible for the biotransformation for endogenous compounds (skatole, sex hormones) as well as exogenous compounds (food components). Both types of compounds are highly expressed in the liver and less in the kidney and adipose tissue. *CYP2A19* transcription is controlled by the CAR transcription factor [78]. Its human orthologue, *CYP2A6* is controlled by CAR, PXR, glucocorticoid receptor (GR), estrogen receptor α, HNF_4_ α, and PGC-1*α* [79]. Also, the constitutive hepatic expression of *CYP2A6* in mice is governed by an interplay between HNF_4_ α, CCAAT-box/enhancer binding protein (C/EBP α, C/EBP β) and octamer transcription factor-1 (Oct-1) [80]. Previously, a positive correlation between mRNA and protein levels for *CYP2A19* gene was observed [81]. Unlike other CYP 450 genes, *CYP2A19* plays a less important role in the xenobiotics’ metabolism but is involved in the reaction of cells to stress, Nrf-2, being also involved in *CYP2A19* transcription [82]. The *CYP2A19* gene is probably highly polymorphic compared to the *CYP2A6* gene [83], and an extensive interindividual variation of its product could occur. Previous studies revealed that duck P450 orthologues of the mammalian *CYP2A6* and CYP3A4 are involved in AFB1 bioactivation into its epoxide form [84]. Unlike these results, in the present study, no significant changes of *CYP2A19* gene expression were noticed in the E1 and E2 groups, probably due to the high level of expression of this gene in piglet liver. For now, it is difficult to explain why the co-exposure of both mycotoxins and the mixture of grapeseed and sea buckthorn meal decreased the expression of *CYP2A19.* However, this decrease of expression diminished the risk of generation of toxic metabolites.

In pig kidney, the expression of *CYP2A19* is lower compared to that found in liver [79]. Probably due to this lower expression, animal exposure to the mixture of grapeseed and sea buckthorn meal generated an upregulation of Nrf-2 induced *CYP2A19* gene expression due to the luteolin [85] and ferulic acid [86] content.

On the other hand, there is evidence that only two transcription factors, i.e., chick ovalbumin upstream promoter transcription factor (COUP-TF1) and hepatocyte nuclear factor (HNF-1), are involved in the regulation of *CYP2E1* transcription in pigs [87]. *CYP2E1*, like other xenobiotic-metabolizing P450s, is mainly located in the membrane of the endoplasmic reticulum (ER) and can be induced under a variety of metabolic or nutritional conditions. ER stress can be induced by metabolic stress, which is caused by overload of protein/lipid biosynthesis, and oxidative stress, which could trigger the evolutionarily conserved complex homeostatic signaling pathway known as the unfolded protein response (UPR) [88].

It is likely that the level of *CYP2E1* mRNA was approximately the same in the E1 and control groups due to the antagonistic actions of palmitic acid [89], linoleic, and α-linolenic acids [90] that increased this gene transcription and the actions vanillic and p-coumaric acids which decreased it [65].

Recently, it was proved that OTA-containing feed altered the intestinal microbiota in ducks, affecting the cecum microbiota diversity and composition as well as the intestinal barrier. As a result, Gram-negative bacterial-derived lipopolysaccharides entered the blood and liver, causing liver inflammation [91]. In the case of immune-mediated liver injury, the expression of *CYP2E1* was decreased [92]. This situation could occur in the E2 group. It is likely that the cumulative effects of the two mycotoxins and dietary by-products decreased the expression of *CYP2E1* in liver of the E3 group.

In the kidney, free fatty acids, such as palmitate, oleate, and linoleate, are stored in the nephron [93], and these acids probably increased the expression of the *CYP2E1* gene in the kidneys of the E1 group compared to the control level. According to Pfohl-Leszkowicz and Manderville [25], OTA forms adduct with DNA, generating renal genotoxicity and carcinogenesis. It is likely that high levels of OTA stimulated *CYP2E1* gene expression in the kidneys of the E2 group compared to the control level. In the E3 group, it appears that the coadministration of the two mycotoxins and dietary byproducts had antagonistic effects, with the expression of *CYP2E1* gene returning to the control level. Moreover, histological evaluation for the E3 group showed that the byproduct mixture derived from grapeseed and sea buckthorn oil mitigated the harmful damage produced by aflatoxin B1 and ochratoxin A at the hepatic and renal level in piglets after weaning. *CYP2E1*, like other xenobiotic-metabolizing P450s, is mainly located in the membrane of the ER and can be induced under a variety of metabolic or nutritional conditions [89]. The regulation of the *CYP2E1* gene in the E1 group was probably due to the hydroxylation of coumarin-derived compounds that were catalyzed by CYP2A enzymes, which are considered to be specific indicators for the presence of CYP2 enzymes [94], with the p-coumaric acid being present in grapeseed and sea buckthorn byproducts.

In the case of pigs, very little is known about the presence of CYP3As enzymes in the renal tissue, and nothing is known about their inducibility [95]. Several genes have been identified in the CYP3A subfamily of mammals (for example, five in rat and four in human), but the expression of these genes in renal tissues has been poorly investigated [96]. In terms of gene expression, Ayed-Boussema et al. (2012) [63] and Gonzalez-Arias et al. [97] described an increase of expression levels in all cytochromes assayed (CYP3A4, 2B6, 3A5, and 2C9) in a primary human hepatocyte culture. Previous studies have reported various results regarding the effects of AFB1 and OTA in primary cultured human hepatocytes in which increasing concentrations of these mycotoxins clearly induced CYP3A4 and CYP2B6 mRNA levels in a dose-dependent manner [63].

In contrast, it has been found that in the presence of OTA and AFB1 in liver (Figure 3, group E2), the *CYP3A29* expression level is decreased compared to the control level, perhaps due to activation of the AhR [98]. These data differ from those of Zepnik et al. [99], who reported an increase of OTA hydrolysis by microsomal enzymes from rat liver, specifically for P450 3A1/2 and 3A4, suggesting that this gene expression is modulated in a species-dependent manner.

In some cases, the inhibition of P450 enzymes by polyphenols may have a chemo-preventive effect due to the potential activation of carcinogens by P450 enzymes within the course of their natural metabolic activity. The inhibition of xenobiotic-metabolizing Phase I enzymes could be one target of the chemo-preventive effects of naturally occurring polyphenols.

The increase of *CYP4A24* observed in liver could be a physiological response in the unusual context of aberrant lipid accumulation and absence of *CYP2E1* activity, due to the fact that *CYP2E1* and CYP4A are inducible hepatic microsomal cytochromes *P*-450 involved in hydroxylation of fatty acids, and both can initiate the auto-propagative process of lipid peroxidation. They might be complementary, leading to interactions in the regulation of the individual enzymes [100]. It is therefore clear that CYP4A proteins are key intermediaries in an adaptive response to perturbation of hepatic lipid metabolism [101]. The decreased *CYP4A24* level in the kidney probably leads to the toxic effects generated in liver due to the mycotoxin-contaminated diet, which means that *CYP4A24* regulates hepatic ER stress [102,103].

In the present study, the addition of a mixture of grapeseed and sea buckthorn meal by-products increased expression levels in the kidney, which would be expected to favor the elimination processes and maintenance of the balance of intracellular substances [104]. Moreover, OTA was absorbed in the intestine where the multidrug resistance protein 2 (*MRP2* gene) plays an important role, acting as a xenobiotic outward transporter to reduce the oral bioavailability and the toxin load to organs and, thereby, OTA toxicity. Once OTA reaches the bloodstream, it can reach other organs such as liver, and the MRP2 transporter is again a key primary active transporter involved in anionic conjugate and xenobiotic extrusion into the extracellular space which contributes to bile formation and the subsequent elimination of the toxin [97,105]. Also, the MRP2 transporter is present in the apical membranes of enterocytes, kidney-proximal tubules, and other cells [105]. OTA toxicity has been attributed to its isocoumarin moiety, and it is well known that OTA is inactivated or bioactivated by cytochrome P450 enzymes [29]. Previously, the presence of OTA in feed was linked to the development of nephrotoxicity, which, in rats, has been associated with renal adenomas and kidney tumors [97]. In the present study, a decrease of *MRP2* expression in the liver was found, indicating an impairment of the secretion of mycotoxins in the E2 group.

In rats, OTA was observed to be excreted 15% less in the proximal tubules of the kidney, while the proximal tubular transport of amino acids was not impaired [97,106]. Therefore, the decrease of *MRP2* in liver found in this study could be the mechanism through which mycotoxins reach high percentages of bioavailability in vivo. In this way, the AFB1 and OTA exposure of piglets would be magnified, contributing to the hepatotoxicity.

Considering the nephrotoxic potential of OTA and AFB1, the decrease of the *MRP2* gene product may also have a major impact on the proximal tubule, leading to a decreased capacity to eliminate OTA [97]. However, further studies are needed on the AFB1 and OTA transporter mechanism to support this hypothesis.

In Phase II of metabolic detoxification, the original xenobiotic compound or the intermediate metabolites modified during Phase I are conjugated in order to be suitable for excretion. Glutathione S transferases (GSTs) and UDP glycurosyltranferases (UGTs) contribute to Phase II processing [107].

In the presence of a mixture of grapeseed and sea buckthorn meal byproducts in pigs feed, the *GSTA1* expression level in liver is significantly increased, possibly by an antioxidant-responsive element (ARE) and β-NF-responsive element (β-NF-RE), respectively, which, in the presence of phenolic antioxidants, activate the GST isoforms without the need for aryl hydrocarbon (Ah) receptors [108]. Surprisingly, in the study of Ghadiri et al. (2019) [109], the AFB1-mediated mRNA downregulation of *GSTA1* was observed in the cow’s liver in the presence of an antioxidant.

Previous studies [110] showed that OTA and AFB1 compete for the same CYP450 enzymes which represent the bioactivation route of AFB1, with less AFB1-DNA adducts being produced. Due to this competition, AFB1 could probably be conjugated with reduced glutathione in a reaction catalyzed by GST enzymes, with their codding genes being upregulated. AFB1 could be involved in other types of Phase II reactions, i.e., glucuronidation and sulfatation, whereas OTA is mainly conjugated with reduced glutathione [72]. Moreover, in response to concomitant administration in the pigs, the feed of two mycotoxins (AFB1 and OTA) increased the generation of the oxidative stress biomarkers. Therefore, defense mechanisms were activated, promoting adaptation and survival in response to oxidative stress [111]. For example, ROS and oxidants could activate the transcription of GST isoforms through ARE [108], as observed in both the liver and kidneys through an increase in the expression level of the *GSTA1* gene.

## 4. Conclusions

Our data revealed the existence of differences between piglet’s kidney and liver regarding the reaction against both mycotoxins and by-products used in this study. Generally, the by-products with antioxidant action decreased the expression of the analyzed CYPs mRNA in liver and increased them in kidney. Also, in both organs, the co-exposure of piglets to OTA and AFB1 generated an increase or a decrease of gene expression dependent on the gene type. The inclusion of grapeseed and sea buckthorn meal in the diet of OTA and AFB1-intoxicated pigs decreased the CYP P450 gene expression, suggesting the decrease of bioactivation of these mycotoxins, probably resulting in a diminished toxicity in both organs, as the histological studies have revealed.

These findings suggest that grapeseed and sea buckthorn meal waste represent a promising source in counteracting the harmful effect of ochratoxin A and aflatoxin B1. Although additional work is needed to unravel the mechanisms by which grapeseed and sea buckthorn byproducts affects AFB1 and OTA biotransformation, and hence the generation of toxic metabolites, the protective effects seem to be at least partly mediated by the enhancement of the antioxidant defense at the liver and kidney level.

## 5. Materials and Methods

### 5.1. Experimental Design and Samples Collection

Forty cross-bred TOPIGS-40 hybrid (♀ Large White × Hybrid (Large White × Pietrain) × ♂ Talent, mainly Duroc) piglets after weaning with an average body weight of 9.11 ± 0.03 kg were assigned to three experimental groups (E1, E2, E3) and one control group (C), housed in pens (two replicates of five pigs per pen per treatment) and fed with experimental diets for 30 days. Feed and water were offered ad libitum during the experiment. The basal diet was served as a control and contained normal compound feed for starter piglets without mycotoxin (corn 68.46%, soya meal 19%, corn gluten 4%, milk replacer 5%, L-lysine 0.3%, DL-methionine 0.1%, limestone 1.57%, monocalcium phosphate 0.35%, salt 0.1%, choline premixes 0.1%, and 1% vitamin-mineral premixes). The experimental groups were fed as follows: E1—basal diet plus a mixture (1:1) of two byproducts (grapeseed and sea buckthorn meal) in a percentage of 5% by replacing corn and soya bean meal; E2—the basal diet artificially contaminated with mycotoxins (a mixture of 62 ppb aflatoxin B1- AFB1 and 479 ppb ochratoxin A-OTA); and E3—basal diet containing 5% of the mixture (1:1) of grapeseed and sea buckthorn meal and contaminated with the mix of AFB1 and OTA. The mixture of OTA and AFB1 mycotoxins was kindly provided by Dr. Boudra and Dr. Morgavi from I. N. R. A, Centre of Clermont Ferrand, and was produced by the cultivation of *Aspergillus flavus* and *Aspergillus ochraceous* on wheat as already described by Boudra et al. [112]. The contaminated material obtained was incorporated into the diets for the E2 and E3 groups, resulting in a final concentration of 479 ppb OTA and 62 ppb AFB1. Animals from all experimental groups had free access to the treatment feed and water every day of the experimental period (30 days). The grapeseed meal and sea buckthorn meal were provided by two local commercials, S.C. OLEOMET-S.R.L. and BIOCATINA, Bucharest, Romania. After 4 weeks, the animals were slaughtered with the approval of the Ethical Committee of the National Research-Development Institute for Animal Nutrition and Biology, Balotești, Romania (Ethical Committee no. 118/02.12.2019) and in accordance with the Romanian Law 206/2004 and the EU Council Directive 98/58/EC for handling and protection of animals used for experimental purposes. At the end of the experimental period of this study, the productive parameters, weight, and feed consumption were measured. Liver and kidney samples were collected from four animals per group and perfused with ice-cold saline solution to remove blood. Fragments of ~50 mg from the right liver lobe and renal cortex (three from each) were collected in RNAlater Stabilization Reagent (Qiagen, Germantown, Maryland) and then stored at −80 °C until RNA isolation step.

Due to ethical reasons, maximizing the use of each animal, minimizing the loss of animals, and statistical analysis, the number of individuals was reduced as much as scientifically possible. Good science and good experimental design help to reduce the number of animals used in any research study, allowing scientists to gather data using the minimum number of animals required [113].

### 5.2. Feed Characterization

Feed diets were analyzed for basal chemical composition (dry matter, crude protein, crude fat, crude fiber, and ash) according to the International Standard Organization methods (SR ISO 6496/2001, Standardized Bulletin (2010). http://www.asro.ro (accessed on 13 February 2021)). Bioactive compounds from byproducts meals, such as polyphenols, polyunsaturated fatty acids (PUFA), and minerals, were determined by Folin-Ciocalteu reaction, HPLC-UV-Vis, and gas chromatography as described by the authors of [113,114]. Antioxidant activity was determined by the DPPH method as described previously by the authors of [115].

### 5.3. Plasma Biomarkers Analysis

On day 30, blood samples were aseptically collected from fasted piglets. Markers that reflect the functionality of liver (aspartate transaminase-AST, alanine transaminase-ALT, gamma glutamyl transferase-GGT, total protein, alkaline phosphatase-AKL), and kidneys (albumin, creatinine) were determined after blood centrifugation using a Clinical Chemistry benchtop analyser Horiba Medical—ABX Pentra 400, (Irvine, CA, USA).

### 5.4. Light Microscopy Examination

Liver and kidney biopsies were fixed in 4% phosphate-buffered formaldehyde solution, dehydrated, clarified, and included in paraffin blocks. The 5 μm sections were processed routinely for hematoxylin-eosin and Gomori trichrome (Leica Biosystems, 38016SS1, Nussloch, Germany) staining, respectively, according to Leica’s protocol. Microscopic sections were analyzed with an Olympus BX43 microscope equipped with a digital camera Olympus XC30. The histopathological alterations of liver and kidney were graded by the severity of lesions as belonging to grades 1–4, as previously described [116]. For liver, grade 1: Normal aspect; grade 2: Normal hepatocytes, slight dilated sinusoids and congestion; grade 3: Vacuolated hepatocytes, dilated sinusoids and congestion; moderate collagen proliferation; grade 4: Necrosis, inflammatory infiltrates, collagen proliferation. For kidney, grade 1: Normal aspect; grade 2: Slight tubular/glomerular injuries, inflammation, and collagen proliferation; grade 3: Mild tubular/glomerular injuries, inflammation, and collagen proliferation; grade 4: Marked tubular/glomerular injuries, inflammation, and collagen proliferation. A “mean assessment value” (MAV) was calculated as a mean of all data per experimental group.

### 5.5. RNA Isolation

The isolation of total RNA was performed from 10 mg of tissue using the RNeasy Plus Universal Mini Kit (Qiagen) following the manufacturer’s protocol. Moreover, it included the On-column DNase digestion step. After RNA isolation, aliquots were made in order to prevent degradation induced by freeze-thaw cycles. The concentration and purity of total RNA were determined using NanoDrop 8000 spectrophotometer (Thermo Scientific, Wilmington, DE, USA).

### 5.6. RNA Integrity Number (RIN)

RIN values of the RNA samples were determined using the Agilent RNA 6000 Nano Kit (Agilent, Santa Clara, CA, USA) and Agilent 2100 Bioanalyzer using the manufacturer’s protocol. Samples with RIN values smaller than 8 were not included in further analysis, and the isolation steps were repeated.

### 5.7. Reverse Transcription

For cDNA synthesis, 1000 ng of total RNA was subjected to reverse transcription using iScript cDNA synthesis kit (Bio-Rad, Hercules, CA, USA). A 4 µL reaction mix and 1 µL reverse transcriptase were mixed with 1 µL RNA samples and completed with RNase free water to a total volume of 20 µL. The final concentration of RNA was 1000 ng per reaction. The reaction was performed using a Veriti 96-Well thermal cycler (Applied Biosystems, Foster City, CA, USA) with the following program: One cycle of 25 °C for 5 min, one cycle of 42 °C for 30 min and one cycle of 85 °C for 5 min. The concentration and purity of the cDNA samples was determined using NanoDrop 8000 spectrophotometer (Thermo Scientific).

### 5.8. Primer Design

Because of the lack of data regarding genes involved in the hepato-nephrotoxicity in the mycotoxin exposure of weaned pigs, primer sequences (Table 7) were designed in silico using Primer3Plus [59] and verified by BLAST program [117]. Those with the highest specificity for the target sequence were selected in order to amplify the *CYP1A2*, *CYP2A19*, *CYP2E1*, *CYP3A29*, *CYP4A24*, *MRP2*, and *GSTA1* genes and three reference genes encoding for TATA-box binding protein, ribosomal protein L4, and beta-2-microglobulin in *Sus scrofa*. The annealing temperatures of the primers were determined by temperature gradient PCR.

### 5.9. Real-Time PCR

The Real-Time PCR reaction was carried out on the iCycler iQ Real-Time PCR Detection System (Bio-Rad) using iQ SYBR Green SuperMix (Bio-Rad). In a 96-well plate, 1 µL of 100 ng/µL cDNA, 12.5 µL iQ SYBR Green SuperMix (Bio-Rad), 0.5 µL of 20 pmol/µL forward primer, 0.5 µL of 20 pmol/µL reverse primer, and 10.5 µL of MilliQ water were added. The total volume was 25 µL. The amplification program was comprised of 1 cycle of 95 °C for 5 min, 45 cycles of 95 °C for 30 s 55/56 °C for 30 s, 72 °C for 45 s, and 85 cycles of 55 °C, with an increase of set point temperature by 0.5 °C per cycle for 10 s. The samples were run, and the threshold cycles (Ct) values were recorded. Melting curves were also performed.

### 5.10. Data Analysis

The Ct values were processed as stated in “The MIQE Guidelines: Minimum Information for Publication of Quantitative Real-Time PCR Experiments” [118] using OpenOffice Calc according to the 2-ΔΔCt method described by Livak and Schmittgen (2001) [119]. The reference genes (TBP, RPL4, and B2M) were chosen in order to be stably expressed across different tissue types and treatments on swine specimens [120,121]. The relative expression value (2^−ΔΔCt^) was obtained by normalization, subtracting the arithmetic mean of the reference genes from each gene of interest. Technical replicates were averaged before statistical analysis. The data are illustrated as average values of the groups (n = 4) ± standard error deviation of the mean (STDEV). All data were statistically analyzed using a one-way ANOVA method performed with GraphPad Prism 3.03 software (GraphPad Software, La Jolla, CA, USA). Post-hoc comparisons between all groups were run using the Bonferroni test. The statistical significance (*p* value) was presented for all groups in contrast to the Control group (C).

## Figures and Tables

**Table 1:** Chemical composition of grapeseed and sea buckthorn.

Byproducts	DM (103 °C) %	CP (%)	EE (%)	Ash (%)	Carbohydrates (mg/g)			
					Fructose	Glucose	Sucrose	Maltose
Sea buckthorn meal	84.48	15.67	10.28	2.75	9.78	7.68	8.03	0.43
Grapeseed meal	90.85	11.32	6.17	3.34	8.34	5.60	3.49	0.54

DM = Dry matter; CP = Crude protein; EE = Fat (ethyl esters).

**Table 2:** Fatty acid composition of grapeseed and sea buckthorn (g FAME/100 gTotal FAME).

Saturated Fatty Acids	Sea Buckthorn Meal	Grapeseed Meal	Unsaturated Fatty Acids	Sea Buckthorn Meal	Grapeseed Meal
Butiric (4:0)	0.07	0.12	Miristoleic (14:1)	0.09	0.05
Caproic (6:0)	0.07	0.16	Pentadecenoic (C15:1)	0.00	0.08
Caprilic (10:0)	0.20	0.18	Palmitoleic (C16:1n-7)	14.28	0.33
Capric (10:0)	0.24	0.17	Heptadecenoic (17:1)	0.05	0.00
Lauric (12:0)	0.03	0.03	Oleic cis (C18:1n-9)	31.07	14.66
Miristic (C14:0)	0.93	0.59	Linoleic cis (C18:2n-6)	18.59	67.35
Pentadecanoic (15:0)	0.17	0.07	Linolenic (C18:3n-6)	0.00	0.04
Palmitic (C16:0)	24.32	9.69	α -Linolenic (C18:3n-3)	6.09	0.94
Heptadecanoic (17:0)	0.12	0.09	Octadecatetraenoic (C18:4n-3)	0.28	0.23
Stearic (C18:0)	2.00	3.56	Eicosadienoic (C20:2n-6)	0.44	0.21
			Arachidonic (C20:4n-6)	0.00	0.20
			Eicosapentaenoic (C20:5n-3)	0.19	0.26
			Lignoceric (C24:0)	0.25	0.31
			Nervonic (C24:1n-9)	0.00	0.13
Other fatty acids	0.51	0.55			
Σ SFA	28.40	14.98			
Σ UFA	71.09	84.47			
Σ MUFA	45.49	15.25			
Σ PUFA	25.60	69.23			
SFA/UFA	0.399	0.177			
PUFA/MUFA	0.563	4.541			
Linoleic/α-Linolenic	3.05	71.64			

FAME = Fatty Acid Methyl Esters; SFA = Saturated fatty acids; UFA = Unsaturated fatty acids; MUFA = Monounsaturated fatty acids; PUFA = Polyunsaturated fatty acids.

**Table 3:** Flavonoids and phenolic acids composition of byproducts.

Flavonoids (mg/g)	Sea Buckthorn Meal	Grapeseed Meal	Phenolic Acids (mg/g)	Sea Buckthorn Meal	Grapeseed Meal
Catechin	0.119	0.378	Vanillic acid	0.008	0.062
Epicatechin	0.397	0.271	Caffeic acid	0.003	0.001
Rutin	0.021	0.009	P-Coumaric acid	0.041	0.005
Quercetin	0.019	0.005	Ferulic acid	0.500	0.063
Luteolin	0.077	0.008			

**Table 4:** Minerals composition of byproducts.

Macroelements (%)	Sea Buckthorn Meal	Grapeseed Meal	Microelements (ppm)	Sea Buckthorn Meal	Grapeseed Meal
Calcium (Ca)	0.04	0.79	Copper (Cu)	7.26	15.46
Phosphor (P)	0.34	0.35	Iron (Fe)	625.77	89.65
Natrium (Na)	0.117	0127	Manganese (Mn)	22.34	18.27
Kalium (K)	1.69	0.89	Zinc (Zn)	21.90	18.66
Magnesium (Mg)	0.127	0.005			

**Table 5:** Biomarkers of liver and kidney function in plasma.

	Control		E1		E2		E3	
	Mean	SEM	Mean	SEM	Mean	SEM	Mean	SEM
Total protein (g/dL)	5.34	0.10	5.08	0.82	5.05	0.18	5.41	0.85
Bilirubin (mg/dL)	0.35	0.04	0.43	0.09	0.31	0.02	0.30	0.06
ALAT (U/L)	49.44	2.36	48.33	1.49	47.22	1.95	50.24	3.56
ASAT (U/L)	38.50	2.92	41.24	3.98	39.96	3.05	41.04	3.28
ALP (U/L)	247.58 ^a^	11.1	279.88 ^ac^	28.3	311.44 ^bc^	25.4	273.22 ^ac^	15.9
GGT (U/L)	26.3 ^a^	2.55	26.67 ^ac^	2.27	34.02 ^bc^	3.34	29.92 ^ac^	1.88
Albumin (g/L)	3.00	0.00	3.00	0.00	3.02	0.01	3.18	0.02
Creatinine (mg/dL)	0.92	0.33	0.96	0.03	0.94	0.03	0.87	0.05

ALAT = alanine transaminase; ASAT = aspartate transaminase; ALK = alkaline phosphatase; GGT = gamma glutamyl transferase; SEM = standard error of mean; ^a,b,c^ Mean values within a row with unlike superscript letters were significantly different (*p* < 0.05).

**Table 6:** The morphometric analysis of the structural injuries in liver and kidney of experimental groups.

MAV	Control Group	E1 Group	E2 Group	E3 Group
Liver	1	1	3.5 ± 0.55 ***	2.5 ± 0.55 ***/^
Kidney	1	1	3.7 ± 0.52 ***	2.3 ± 0.52 ***/^^

One-Way ANOVA test. * (All groups vs. Control group; *** *p* < 0.001). ^ (E3 group vs. E2 group; ^ *p* < 0.05; ^^ *p* < 0.01). MAV = Mean assessment value.

**Table 7:** Primers for Real-Time PCR analysis.

GenBank Accession Number	Gene	PCR Product Length (bp)	Primer Name	Primer Sequence
XM021085497	TATA-box binding protein	124	tbp-F	5′-GATGGACGTTCGGTTTAGG-3′
			tbp-R	5′-AGCAGCACAGTACGAGCAA-3′
XM005659862	ribosomal protein L4	122	rpl4-F	5′-CAAGAGTAACTACAACCTTC-3′
			rpl4-R	5′-GAACTCTACGATGAATCTTC-3′
NM213978	beta-2-microglobulin	172	b2m-F	5′-CCGCCCCAGATTGAAATTGA-3′
			b2m-R	5′-GCTTATCGAGAGTCACGTGC-3′
NM001159614	cytochrome P450, family 1, subfamily A, polypeptide 2	173	cyp1a2-F	5′-CTCTTCCGACACACCTCCTT-3′
			cyp1a2-R	5′-AATCTCTCTGGCCGGAACTC-3′
NM214417	cytochrome P450 2A19	174	cyp2a19-F	5′-CTCATGAAGATCAGCCAGCG-3′
			cyp2a19-R	5′-GCCATAGCCTTTGAAGAGCC-3′
XM005657509	cytochrome P450, family 2, subfamily E, polypeptide 1	150	cyp2e1-F	5′-ACCTCATTCCCTCCAACCTG-3′
			cyp2e1-R	5′-CTGGCTTAAACTTCTCCGGC-3′
NM214423	cytochrome P450 3A29	205	cyp3a29-F	5′-ATTGCTGTCTCCGACCTTCA-3′
			cyp3a29-R	5′-TGGGTTGTTGAGGGAATCGA-3′
XM021096706	cytochrome P450 4A24	157	cyp4a24-F	5′-CTCTATCCGCCAGTACCAGG-3′
			cyp4a24-R	5′-ATGGGTCAAACTCCTCTGGG-3′
XM021073710	ATP binding cassette subfamily C member 2	172	mrp2-F	5′-AGCAGTACACCGTTGGAGAA-3′
			mrp2-R	5′-ATCACCCCAACACCTGCTAA-3′
NM214389	glutathione S-transferase alpha 1	186	gsta1-F	5′-GCCCATGGTTGAGATTGACG-3′
			gsta1-R	5′-TTTTCATTGGGTGGGCACAG-3′

## Data Availability

The data presented in this study are available on request from the corresponding author. The data are not publicly available due to privacy reason.

## References

[1] Liew W.-P.-P., Mohd-Redzwan S. (2018). Mycotoxin: Its Impact on Gut Health and Microbiota. Front. Cell. Infect. Microbiol..

[2] Desjardins A.E., Hohn T.M. (2007). Mycotoxins in Plant Pathogenesis. Mol. Plant.-Microbe Interact..

[3] Riley R.T., Pestka J., Diaz D. (2005). Mycotoxins: Metabolism, mechanisms and biochemical markers. The Mycotoxin Blue Book.

[4] Feddern V., Dors G.C., Tavernari F., Mazzuco H., Cunha J.A., Krabbe E.L., Scheuermann G.N. (2013). Aflatoxins: Importance on animal nutrition. Aflatoxins Recent Advances and Future Prospects.

[5] Medina A., Akbar A., Baazeem A., Rodriguez A., Magan N. (2017). Climate change, food security and mycotoxins: Do we know enough?. Fungal Biol. Rev..

[6] Pierron A., Alassane-Kpembi I., Oswald I.P. (2016). Impact of mycotoxin on immune response and consequences for pig health. Anim. Nutr..

[7] Streit E., Schatzmayr G., Tassis P., Tzika E., Marin D., Taranu I., Tabuc C., Nicolau A., Aprodu I., Puel O. (2012). Current situation of mycotoxin contamination and co-occurrence in animal feed—Focus on Europe. Toxins.

[8] Gruber-Dorninger C., Jenkins T., Schatzmayr G. (2020). Global Mycotoxin Occurrence in Feed: A Ten-Year Survey. Toxins.

[9] Marin D.E., Motiu M., Taranu I. (2015). Food Contaminant Zearalenone and Its Metabolites Affect Cytokine Synthesis and Intestinal Epithelial Integrity of Porcine Cells. Toxins.

[10] Alassane-Kpembi I., Kolf-clauw M., Gauthier T., Abrami R., Abiola F.A., Oswald I.P., Puel O. (2013). New insights into mycotoxin mixtures: The toxicity of low doses of Type B trichothecenes on intestinal epithelial cells is synergistic. Toxicol. Appl. Pharmacol..

[11] Broom L.J., Wood M., Park E., Kingdom U. (2015). Organic acids for improving intestinal health of poultry. Worlds Poult. Sci. J..

[12] Filazi A., Sireli U.T., Mehdi R.-A. (2012). Occurrence of aflatoxins in food. Aflatoxins: Recent Advances and Future Prospects.

[13] Seetha A., Munthali W., Msere H.W., Swai E., Muzanila Y., Sichone E., Tsusaka T.W., Rathore A., Okori P. (2017). Occurrence of aflatoxins and its management in diverse cropping systems of central Tanzania. Mycotoxin Res..

[14] Ismail A., Gonçalves B.L., de Neeff D.V., Ponzilacqua B., Coppa C.F.S.C., Hintzsche H., Sajid M., Cruz A.G., Corassin C.H., Oliveira C.A.F. (2018). Aflatoxin in foodstuffs: Occurrence and recent advances in decontamination. Food Res. Int..

[15] Negash D. (2018). Citation: Negash D (2018) A Review of Aflatoxin: Occurrence, Prevention, and Gaps in Both Food and Feed Safety. J. Appl. Microbiol. Res..

[16] Arroyo-Manzanares N., Rodríguez-Estévez V., Arenas-Fernández P., García-Campaña A.M., Gámiz-Gracia L. (2019). Occurrence of Mycotoxins in Swine Feeding from Spain. Toxins.

[17] Nazhand A., Durazzo A., Lucarini M., Souto E.B., Santini A. (2020). Characteristics, Occurrence, Detection and Detoxification of Aflatoxins in Foods and Feeds. Foods.

[18] Dhama K., Singh K.P. (2014). Aflatoxins- Hazard to Livestock and Poultry Production: A Review. J. Immunol. Immunopathol..

[19] Devreese M., De Backer P., Croubels S. (2013). Overview of the most important mycotoxins for the pig and poultry husbandry Overzicht van de meest belangrijke mycotoxines voor de varkens-en pluimveehouderij. Vlaams Diergeneeskd. Tijdschr..

[20] Jatfa J.W., Wachida N.M., Ijabo H.M., Adamu S.S. (2018). Aflatoxicosis Associated with Swine Stillbirth in the Piggery Farm University of Agriculture Makurdi. Curr. Trends Biomedical. Eng. Biosci..

[21] Lee H.S., Lindahl J., Nguyen-Viet H., Khong N.V., Nghia V.B., Xuan H.N., Grace D. (2017). An investigation into aflatoxin M1in slaughtered fattening pigs and awareness of aflatoxins in Vietnam. BMC Vet. Res..

[22] Wilfred E.G., Dungworth D.L., Moulton J.E. (1968). Pathologic Effects of Aflatoxin in Pigs. Vet. Pathol..

[23] Dilkin P., Zorzete P., Mallmann C.A., Gomes J.D.F., Utiyama C.E., Oetting L.L., Corrêa B. (2003). Toxicological effects of chronic low doses of aflatoxin B1 and fumonisin B1-containing Fusarium moniliforme culture material in weaned piglets. Food Chem. Toxicol..

[24] Obuseh F.A., Jolly P.E., Jiang Y., Shuaib F.M.B., Waterbor J., Ellis W.O., Piyathilake C.J., Desmond R.A., Afriyie-Gyawu E., Phillips T.D. (2010). Aflatoxin B1 albumin adducts in plasma and aflatoxin M1 in urine are associated with plasma concentrations of vitamins A and E. Int. J. Vitam. Nutr. Res..

[25] Pfohl-leszkowicz A., Manderville R.A. (2012). An Update on Direct Genotoxicity as a Molecular Mechanism of Ochratoxin A Carcinogenicity. Chem. Res. Toxicol..

[26] Arbillaga L., Azqueta A., Van Delft J.H.M., López A., Cerain D. (2007). In vitro gene expression data supporting a DNA non-reactive genotoxic mechanism for ochratoxin A. Toxicol. Appl. Pharmacol..

[27] Asrani R.K., Patial V., Thakur M., Porter D. (2016). Ochratoxin A: Possible mecanisms of toxicity. Ochratoxins-Biosynthesis, Detection and Toxicity.

[28] Marin D.E., Braicu C., Gras M.A., Pistol G.C., Petric R.C., Neagoe I.B., Palade M., Taranu I.S.C. (2017). Low level of ochratoxin A affects genome-wide expression in kidney of pig. Toxicon.

[29] Ringot D., Chango A., Schneider Y.J., Larondelle Y. (2006). Toxicokinetics and toxicodynamics of ochratoxin A, an update. Chem. Biol. Interact..

[30] Kőszegi T., Poór M. (2016). Ochratoxin a: Molecular interactions, mechanisms of toxicity and prevention at the molecular level. Toxins.

[31] Bayman P., Baker J.L. (2006). Ochratoxins: A global perspective. Mycopathologia.

[32] Peeradon T., Tichakorn S., Anupon T., Supatra P. (2020). Modulation of Edible Plants on Hepatocellular Carcinoma Induced by Aflatoxin B. Phytochemicals in Human Health.

[33] Guyonnet D., Belloir C., Suschetet M., Bon A. (2002). Le Mechanisms of protection against aflatoxin B 1 genotoxicity in rats treated by organosulfur compounds from garlic. Carcinogenesis.

[34] Moudgil V., Redhu D., Dhanda S., Singh J. (2013). A review of molecular mechanisms in the development of hepatocellular carcinoma by aflatoxin and hepatitis B and C viruses. J. Environ. Pathol. Toxicol. Oncol..

[35] Li H., Xing L., Zhang M., Wang J., Zheng N. (2018). The Toxic Effects of Aflatoxin B1 and Aflatoxin M1 on Kidney through Regulating L-Proline and Downstream Apoptosis. BioMed. Res. Intern..

[36] Kanora A., Maes D. (2009). The role of mycotoxins in pig reproduction: A review. Vet. Med..

[37] Li X., Zhao L., Fan Y., Jia Y., Sun L., Ma S., Ji C., Ma Q., Zhang J. (2014). Occurrence of mycotoxins in feed ingredients and complete feeds obtained from the Beijing region of China. J. Anim. Sci. Biotechnol..

[38] Svoboda M., Blahová J., Honzlová A., Kalinová J., Macharáčková P., Rosmus J., Mejzlík V., Kúkol P., Vlasáková V., Mikulková K. (2019). Multiannual occurrence of mycotoxins in feed ingredients and complete feeds for pigs in the Czech Republic. Acta. Vet. Brno..

[39] Khoshal A.K., Novak B., Martin P.G.P., Jenkins T., Neves M., Schatzmayr G., Oswald I.P., Pinton P. (2019). Worldwide Finished Pig Feed and Their Combined Toxicity in Intestinal Cells. Toxins.

[40] Ma R., Zhang L., Liu M., Su Y., Xie W., Zhang N. (2018). Individual and Combined Occurrence of Mycotoxins in Feed Ingredients and Complete Feeds in China. Toxins.

[41] Freitas B.V., Mota M.M., Del Santo T.A., Afonso E.R., Silva C.C., Utimi N.B.P., Barbosa L.C.G.S., Vilela F.G., Araújo L.F. (2012). Mycotoxicosis in Swine: A Review. J. Anim. Prod. Adv..

[42] Adunphatcharaphon S., Petchkongkaew A., Greco D., D’Ascanio V., Visessanguan W., Avantaggiato G. (2020). The Effectiveness of Durian Peel as a Multi-Mycotoxin Adsorbent. Toxins.

[43] Agriopoulou S., Stamatelopoulou E., Varzakas T. (2020). Advances in Occurrence, Importance, and Mycotoxin Control Strategies: Prevention and Detoxification in Foods. Foods.

[44] Solís-Cruz B., Hernández-Patlán D., Beyssac E., Latorre J.D., Hernandez-Velasco X., Merino-Guzman R., Tellez G., López-Arellano R. (2017). Evaluation of Chitosan and Cellulosic Polymers as Binding Adsorbent Materials to Prevent Aflatoxin B1, Fumonisin B1, Ochratoxin, Trichothecene, Deoxynivalenol, and Zearalenone Mycotoxicoses Through an In Vitro Gastrointestinal Model for Poultry. Polymers.

[45] Čolović R., Puvača N., Cheli F., Avantaggiato G., Greco D., Đuragić O., Kos J., Pinotti L. (2019). Decontamination of Mycotoxin-Contaminated Feedstuffs and Compound Feed. Toxins.

[46] Vila-Donat P., Marín S., Sanchis V., Ramos A.J. (2018). A review of the mycotoxin adsorbing agents, with an emphasis on their multi-binding capacity, for animal feed decontamination. Food Chem. Toxicol..

[47] Badr A.N., Abdel-Razek A.G., Youssef M., Shehata M., Hassanein M.M., Amra H. (2021). Natural Antioxidants: Preservation Roles and Mycotoxicological Safety of Food. Egypt. J. Chem..

[48] Gugliandolo E., Peritore A.F., D’Amico R., Licata P., Crupi R. (2020). Evaluation of Neuroprotective Effects of Quercetin against Aflatoxin B1-Intoxicated Mice. Animals.

[49] Antonissen G., Devreese M., De Baere S., Martel A., Van Immerseel F., Croubels S. (2017). Impact of Fusarium mycotoxins on hepatic and intestinal mRNA expression of cytochrome P450 enzymes and drug transporters, and on the pharmacokinetics of oral enrofloxacin in broiler chickens. Food Chem. Toxicol..

[50] Zaragozá C., Villaescusa L., Monserrat J., Zaragozá F., Álvarez-Mon M. (2020). Potential Therapeutic Anti-Inflammatory and Immunomodulatory Effects of Dihydroflavones, Flavones, and Flavonols. Molecules.

[51] Yahfoufi N., Alsadi N., Jambi M., Matar C. (2018). The Immunomodulatory and Anti-Inflammatory Role of Polyphenols. Nutrients.

[52] World Health Organization Mycotoxins. Children’s Health and the Environment. https://www.who.int/ceh/capacity/mycotoxins.pdf.

[53] Perrone G., Ferrara M., Medina A., Pascale M., Magan N. (2020). Toxigenic Fungi and Mycotoxins in a Climate Change Scenario: Ecology, Genomics, Distribution, Prediction and Prevention of the Risk. Microorganisms.

[54] Joubrane K., Mnayer D., El Khoury A., El Khoury A., Awad E. (2020). Co-Occurrence of Aflatoxin B1 and Ochratoxin A in Lebanese Stored Wheat. J. Food Prot..

[55] Ibañez-Vea M., González-Peñas E., Lizarraga E., López de Cerain A. (2012). Co-occurrence of aflatoxins, ochratoxin A and zearalenone in barley from a northern region of Spain. Food Chem..

[56] Gamze N.K., Fatih O., Bulent K. (2015). Co-occurrence of aflatoxins and ochratoxin A in cereal flours commercialised in Turkey. Food Control..

[57] Ozbey F., Kabak B. (2012). Natural co-occurrence of aflatoxins and ochratoxin A in spices. Food Control..

[58] Santos Pereira C., Cunha S., Fernandes J.O. (2019). Prevalent Mycotoxins in Animal Feed: Occurrence and Analytical Methods. Toxins.

[59] Taranu I., Marin D.E., Palade M., Pistol G.C., Chedea V.S., Gras M.A., Rotar C. (2019). Assessment of the e ffi cacy of a grape seed waste in counteracting the changes induced by a fl atoxin B1 contaminated diet on performance, plasma, liver and intestinal tissues of pigs after weaning. Toxicon.

[60] Nilova L., Malyutenkova S. (2018). The possibility of using powdered sea-buckthorn in the development of bakery products with antioxidant properties. Agro. Res..

[61] Balogh K., Hausenblasz J., Weber M., Erdélyi M., Fodor J., Mézes M. (2007). Effects of ochratoxin A on some production traits, lipid peroxide and glutathione redox status of weaned piglets. Acta. Vet. Hung..

[62] Marin D.E., Taranu I. (2015). Ochratoxin A and its effects on immunity. Toxin Rev..

[63] Ayed-Boussema I., Pascussi J.M., Zaied C., Maurel P., Bacha H., Hassen W. (2012). CYP1A2 gene expression in primary cultured human hepatocytes: A possible activation of nuclear receptors. Drug Chem. Toxicol..

[64] Jiang Z., Gu L., Liang X., Cao B., Zhang J., Guo X. (2020). The Effect of Selenium on CYP450 Isoform Activity and Expression in Pigs. Biol. Trace. Elem. Res..

[65] Altay A., Bozoğlu F. (2017). Salvia fruticosa Modulates mRNA Expressions and Activity Levels of Xenobiotic Metabolizing CYP1A2, CYP2E1, NQO1, GPx, and GST Enzymes in Human. Nutr. Cancer.

[66] Rasmussen M.K., Zamaratskaia G., Ekstrand B. (2011). Gender-related Differences in Cytochrome P450 in Porcine Liver-Implication for Activity, Expression and Inhibition by Testicular Steroids. Reprod. Domest. Anim..

[67] Nwafor I.C., Shale K., Achilonu M.C. (2017). Chemical Composition and Nutritive Benefits of Chicory (*Cichorium intybus*) as an Ideal Complementary and/or Alternative Livestock Feed Supplement. Sci. World J..

[68] Dietrich C. (2016). Antioxidant Functions of the Aryl Hydrocarbon Receptor. Stem. Cells. Int..

[69] Kapelyukh Y., Henderson C.J., Scheer N., Rode A., Wolf C.R. (2019). Defining the Contribution of CYP1A1 and CYP1A2 to Drug Metabolism Using Humanized CYP1A1/1A2 and Cyp1a1/Cyp1a2 Knockout Mice. Drug Metab. Dispos..

[70] Sansen S., Yano J.K., Rosamund L., Schoch G.A., Keith J., Stout C.D., Johnson E.F., Sansen S., Yano J.K., Reynald R.L. (2007). Adaptations for the Oxidation of Polycyclic Aromatic. J. Biol. Chem..

[71] Schelstraete W., De Clerck L., Govaert E., Mil J., Devreese M., Deforce D., D. Bocxlaer J., Croubels S. (2019). Characterization of Porcine Hepatic and Intestinal Drug Metabolizing CYP450: Comparison with Human Orthologues from A Quantitative, Activity and Selectivity Perspective. Sci. Rep..

[72] Wen J., Mu P., Deng Y. (2016). Mycotoxins: Cytotoxicity and biotransformation in animal cells. Toxicol. Res. (Camb.).

[73] Liu R., Desai L.P. (2015). Reciprocal regulation of TGF- β and reactive oxygen species: A perverse cycle for fi brosis. Redox Biol..

[74] Muller G.F. (2000). Effect of transforming growth factor- b 1 on cytochrome P450 expression: Inhibition of CYP1 mRNA and protein expression in primary rat hepatocytes. Arch. Toxicol.

[75] Penner N., Woodward C., Prakash C. (2012). Appendix: Drug Metabolizing Enzymes and Biotransformation Reactions. ADME.

[76] Pyo M.C., Shin H.S., Jeon G.Y., Lee K.-W. (2020). Synergistic Interaction of Ochratoxin A and Acrylamide Toxins in Human Kidney and Liver Cells. Biol Pharm Bull..

[77] Zhao H., Chen L., Yang T., Feng Y.L., Vaziri N.D., Liu B.L., Liu Q.Q., Guo Y. (2019). Aryl hydrocarbon receptor activation mediates kidney disease and renal cell carcinoma. J. Transl. Med..

[78] Rasmussen M.K., Zamaratskaia G. (2014). Regulation of Porcine Hepatic Cytochrome P450—Implication for Boar Taint. CSBJ.

[79] Burkina V., Rasmussen M.K., Olünychenko Y., Zamaratskaia G. (2019). Porcine cytochrome 2A19 and 2E1. Basic Clin. Pharmacol. Toxicol..

[80] Pitarque M., Rodriguez-Antona C., Oscarson M., Ingelman-Sundberg M. (2005). Transcriptional regulation of the human CYP2A6 gene. J. Pharmacol. Exp. Ther..

[81] Brunius C., Andersson K., Zamaratskaia G. (2012). Expression and activities of hepatic cytochrome P450 (CYP1A, CYP2A and CYP2E1) in entire and castrated male pigs. Animal.

[82] Yokota S., Higashi E., Fukami T., Yokoi T., Nakajima M. (2011). Human CYP2A6 is regulated by nuclear factor-erythroid 2 related factor 2. Biochem. Pharmacol..

[83] Tanner J., Tyndale R.F. (2017). Variation in CYP2A6 Activity and Personalized Medicine. J. Pers Med..

[84] Diaz G.J., Murcia H.W., Cepeda S.M., Boermans H.J. (2010). The role of selected cytochrome P450 enzymes on the bioactivation of aflatoxin B1 by duck liver microsomes. Avian Pathol..

[85] Kalbolandi S.M., Gorji A.V., Babaahmadi-Rezaei H., Mansouri E. (2019). Luteolin confers renoprotection against ischemia–reperfusion injury via involving Nrf2 pathway and regulating miR320. Mol. Biol. Rep..

[86] Mahmoud A.M., Hussein O.E., Abd El-Twab S.M., Hozayen W.G. (2019). Ferulic acid protects against methotrexate nefrotoxicity via activation of Nrf2/ARE/HO-1 signaling and PPARγ and suppression of NF-kB/NLRP3 inflammasome axis. Food Funct..

[87] Tambyrajah W.S., Doran E., Wood J.D., Mcgivan J.D. (2004). The pig CYP2E1 promoter is activated by COUP-TF1 and HNF-1 and is inhibited by androstenone. Arch. Biochem. Biophys..

[88] Park E.C., Kim S.I., Hong Y., Hwang J.W., Cho G., Cha H., Han J., Yun C., Park S., Jang I. (2014). Inhibition of CYP4A Reduces Hepatic Endoplasmic Reticulum Stress and Features of Diabetes in Mice. Gastroenterology.

[89] Raucy J.L., Lasker J., Ozaki K., Zoleta V. (2004). Regulation of CYP2E1 by Ethanol and Palmitic Acid and CYP4A11 by Clofibrate in Primary Cultures of Human Hepatocytes. Toxicol. Sci..

[90] Sung M., Kim I., Park M., Whang Y., Lee M. (2004). Differential effects of dietary fatty acids on the regulation of CYP2E1 and protein kinase C in human hepatoma HepG2 cells. J. Med. Food.

[91] Wang W., Zhai S., Xia Y., Wang H., Ruan D., Zhou T., Zhu Y., Zhang H., Zhang M., Ye H. (2019). Ochratoxin A induces liver inflammation: Involvement of intestinal microbiota. Microbiome.

[92] Lin Q., Kang X., Li X., Wang T., Liu F., Jia J., Jin Z., Id Y.X. (2019). NF-κB-mediated regulation of rat CYP2E1 by two independent signaling pathways. PLoS ONE.

[93] Meyer C., Nadkarni V., Nadkarni K., Stumvoll M., Gerich J. (1997). Human kidney free fatty acid and glucose uptake: Evidence for a renal glucose-fatty acid cycle. Am. J. Phisiol..

[94] Deng X., Pu Q., Wang E., Yu C. (2016). Celery extract inhibits mouse CYP2A5 and human CYP2A6 activities via different mechanisms. Oncol. Lett.

[95] Ling D., Salvaterra P.M. (2011). Robust RT-qPCR Data Normalization: Validation and Selection of Internal Reference Genes during Post-Experimental Data Analysis. PLoS ONE.

[96] Messina A., Nannelli A., Fiorio R., Longo V., Gervasi P.G. (2009). Expression and inducibility of and CYP2B22, 3A22, 3A29, 3A46 by rifampicin in the respiratory and olfactory mucosa of pig. Toxicology.

[97] Gonzalez-Arias C.A., Crespo-Sempre S., Sanchis V., Ramos A.J. (2015). Modulation of the xenobiotic transformation system and inflammatory response by ochratoxin A exposure using a co-culture system of Caco-2 and HepG2 cells. Food Chem. Toxicol..

[98] Rasmussen M.K. (2020). Porcine cytochrome P450 3A: Current status on expression and regulation. Arch. Toxicol..

[99] Zepnik H., Pa A., Schauer U., Dekant W. (2001). Ochratoxin A-Induced Tumor Formation: Is There a Role of Reactive Ochratoxin A Metabolites?. Toxicol. Sci..

[100] Robertson G., Leclercq I., Farrell G.C., Steatosis C.F.N., Ii S. (2020). Nonalcoholic Steatosis and Steatohepatitis II. Cytochrome. Am. J. Physiol. Gastrointest Liver Pysiol..

[101] Leclercq I.A., Gonzalez F.J., Graham R., Leclercq I.A., Farrell G.C., Field J., Bell D.R., Gonzalez F.J., Robertson G.R. (2000). CYP2E1 and CYP4A as microsomal catalysts of lipid peroxides in murine nonalcoholic steatohepatitis. J. Clin. Investig..

[102] Liu Y., Xu W., Zhai T., You J., Chen Y. (2019). Silibinin ameliorates hepatic lipid accumulation and oxidative stress in mice with non-alcoholic steatohepatitis by regulating CFLAR-JNK pathway. Acta Pharm. Sin. B.

[103] Stading R., Couroucli X., Lingappan K., Moorthy B. (2020). The role of cytochrome P450 (CYP) enzymes in hyperoxic lung injury. Expert Opin. Drug Metab. Toxicol..

[104] Ruan D., Zhu Y.W., Fouad A.M., Yan S.J., Chen W., Zhang Y.N., Xia W.G., Wang S., Jiang S.Q., Yang L. (2018). Dietary curcumin enhances intestinal antioxidant capacity in ducklings via altering gene expression of antioxidant and key detoxification enzymes. Poult. Sci..

[105] Jedlitschky G., Hoffmann U., Kroemer H.K. (2006). Structure and function of the MRP2 (ABCC2) protein and its role. Expert Opin. Drug Metab. Toxicol..

[106] Gekle M., Mildenberger S., Freudinger R., Silbernagl S., Poronnik P., Cook D.I., Young J.A. (1994). pH of endosomes labelled by receptor-mediated and fluid-phase endocytosis and its possible role for the regulation of endocy-totic uptake. Studies in Honour of Karl Julius Ultrich. An Australian Symposium.

[107] Zhang J., Pan Z., Moloney S., Sheppard A. (2014). RNA-Seq Analysis Implicates Detoxification Pathways in Ovine Mycotoxin Resistance. PLoS ONE.

[108] Raghunath A., Sundarraj K., Nagarajan R., Arfuso F., Bian J. (2018). Redox Biology Antioxidant response elements: Discovery, classes, regulation and potential applications. Redox Biol..

[109] Ghadiri S., Spalenza V., Dellafiora L., Badino P., Barbarossa A., Dall C., Nebbia C., Girolami F. (2019). Toxicology in Vitro Modulation of aflatoxin B1 cytotoxicity and aflatoxin M1 synthesis by natural antioxidants in a bovine mammary epithelial cell line. Toxicol. Vitr..

[110] Corcuera L., Vettorazzi A., Arbillaga L., Pérez N., Gloria A., Azqueta A., González-peñas E., García-jalón J.A., López A., Cerain D. (2015). Genotoxicity of Aflatoxin B1 and Ochratoxin A after simultaneous application of the in vivo micronucleus and comet assay. Food Chem. Toxicol..

[111] Shin H.S., Lee H.J., Pyo M.C., Ryu D., Lee K.-W. (2019). Ochratoxin A-Induced Hepatotoxicity through Phase I and Phase II Reactions Regulated by AhR in Liver Cells. Toxins (Basel).

[112] Boudra H.S., Saivin S., Buffiere C., Morgavi D.P. (2013). Short communication: Toxicokinetics of ochratoxin A in dairy ewes and carryover to milk following a single or long-term ingestion of contaminated feed. J. Dairy Sci..

[113] Festing S., Wilkinson R. (2007). The ethics of animal research. Talking Point on the use of animals in scientific research. EMBO Rep..

[114] Taranu I., Braicu C., Marin D.E., Pistol G.C., Motiu M., Balacescu L., Beridan Neagoe I., Burlacu R. (2015). Exposure to zearalenone mycotoxin alters in vitro porcine intestinal epithelial cells by differential gene expression. Toxicol. Lett..

[115] Taranu I., Habeanu M., Gras M.A., Pistol G.C., Lefter N., Palade M., Ropota M., Chedea V.S., Marin D.E. (2017). Assessment of the effect of grape seed cake inclusion in the diet of healthy fattening- finishing pigs. J. Anim. Physiol. Anim. Nutr. (Berl).

[116] Hermenean A., Damache G., Albu P., Ardelean A., Ardelean G., Puiu Ardelean D., Horge M., Nagy T., Braun M., Zsuga M. (2015). Histopatological alterations and oxidative stress in liver and kidney of Leuciscus cephalus following exposure to heavy metals in the Tur River, North Western Romania. Ecotoxicol. Environ. Saf..

[117] Untergasser A., Nijveen H., Rao X., Bisseling T. (2007). Primer3Plus, an enhanced web interface to Primer3. Nucleic Acids Res..

[118] Altschup S.F., Gish W., Pennsylvania T., Park U. (1990). Basic Local Alignment Search Tool 2Department of Computer Science. J. Mol. Biol..

[119] Bustin S.A., Benes V., Garson J.A., Hellemans J., Huggett J., Kubista M., Mueller R., Nolan T., Pfaffl M.W., Shipley G.L. (2009). The MIQE Guidelines: Minimum Information for Publication of Quantitative Real-Time PCR Experiments. Clin. Chem..

[120] Livak K.J., Schmittgen T.D. (2001). Analysis of Relative Gene Expression Data Using Real- Time Quantitative PCR and the 2^−ΔΔCT^ Method. Methods.

[121] Sandercock D.A., Coe J.E., Di P., Edwards S.A. (2017). Research in Veterinary Science Determination of stable reference genes for RT-qPCR expression data in mechanistic pain studies on pig dorsal root ganglia and spinal cord. Res. Vet. Sci..

